# X‐ray video defaecography is superior to magnetic resonance defaecography in the imaging of defaecation disorders

**DOI:** 10.1111/codi.16081

**Published:** 2022-02-17

**Authors:** Eija Pääkkö, Johanna Mäkelä‐Kaikkonen, Hannele Laukkanen, Pasi Ohtonen, Kirsi Laitakari, Tero Rautio, Heljä Oikarinen

**Affiliations:** ^1^ 60664 Department of Diagnostic Radiology Oulu University Hospital Oulu Finland; ^2^ 60664 Division of Gastroenterology Department of Surgery Oulu University Hospital Oulu Finland; ^3^ Medical Research Center Oulu Center of Surgical Research University of Oulu Oulu Finland; ^4^ 60664 Division of Operative Care Oulu University Hospital Oulu Finland; ^5^ The Research Unit of Surgery, Anesthesia and Intensive Care University of Oulu Oulu Finland

**Keywords:** defaecography, MR defaecography, pelvic floor weakness, pelvic organ prolapse

## Abstract

**Aim:**

The aim of this work was to study the technical success and diagnostic capability of magnetic resonance defaecography (MRD) compared with video defaecography (VD).

**Method:**

Sixty four women with defaecation disorders underwent both MRD and x‐ray VD over 1 year. The assessment by two radiologists in consensus was retrospective and blinded. The technical success of straining and evacuation was evaluated subjectively. The presence of enterocele, intussusception, rectocele and dyssynergic defaecation was analysed according to established criteria, with VD as the standard of reference.

**Results:**

It was found that 62/64 (96.9%) VD studies were technically fully diagnostic compared with 29/64 (45.3%) for MRD. The number of partially diagnostic studies was 1/64 (1.6%) for VD versus 21/64 (32.8%) for MRD, with 1/64 (1.6%) (VD) and 14/64 (21.9%) (MRD) being nondiagnostic. Thirty enteroceles were observed by VD compared with seven in MRD with moderate agreement (*κ* = 0.41). Altogether 53 intussusceptions were observed by VD compared with 27 by MRD with poor agreement (*κ* = −0.10 and *κ* = 0.02 in recto‐rectal and recto‐anal intussusception, respectively). Moderate agreement (*κ* = 0.47) was observed in diagnosing rectocele, with 47 cases by VD and 29 by MRD. Dyssynergic defaecation was observed in three patients by VD and in 11 patients by MRD, with slight agreement (*κ* = 0.14).

**Conclusion:**

The technical success and diagnostic capabilities of VD are better than those of MRD. VD remains the method of choice in the imaging of defaecation disorders.

**What does this paper add to the literature?**

This is the first paper to systematically analyse the technical success rate of video defaecography (VD) and magnetic resonance defaecography (MRD), paying special attention to straining and evacuation. VD was fully diagnostic in 96.9% of cases, whereas 21.9% of MRD studies were nondiagnostic due to deficient straining and evacuation.

## INTRODUCTION

Pelvic floor dysfunction is a common debilitating condition especially affecting postmenopausal, multiparous women. Pelvic floor weakness may cause obstructed defaecation syndrome (ODS) and faecal incontinence (FI). Clinical examination is often insufficient in assessing pelvic prolapses, especially enteroceles and sigmoidoceles [1, 2]. X‐ray video defaecography (VD) and dynamic magnetic resonance defaecography (MRD) are the imaging methods used in the study of posterior pelvic compartment disorders. Imaging plays a pivotal role in treatment decisions, altering the choice of treatment and surgical approach in a substantial number of patients [2, 3, 4].

Video defaecography has been the gold standard diagnostic method in imaging of defaecation disorders. It is considered as the ‘functional’ method because patients are in a sitting position during the examination [5]. This position allows complete relaxation of the levator ani and defaecation, which are needed to diagnose defaecation disorders. VD is the traditional and widely available method and is cheaper than magnetic resonance imaging (MRI). However, there is a concern about the possible risks of ionizing radiation, especially when examining younger patients [6].

MRI is an excellent method for imaging soft tissues and it allows multiplanar evaluation of pelvic organs without ionizing radiation. With the development of the technique it is possible to use rapid MR sequences, allowing real‐time dynamic evaluation of defaecation. Nowadays, MRD is performed in closed magnets allowing only supine positioning of the patient. A supine position is not the physiological defaecation position, which may be a substantial disadvantage [6, 7]. However, due to the aforementioned advantages, there has been growing interest in using MRD in patients with defaecation disorders [8, 9, 10].

Several studies have compared these two methods in pelvic organ prolapse and defaecation disorders, with variable results. MRD was found superior to dynamic fluoroscopy in detecting pelvic floor descent and prolapse in women [11]. Based on the results of a retrospective study, it was assessed to be equivalent to VD for abnormalities of the posterior compartment of the pelvic floor [12]. In another study, more enteroceles were detected by MRI compared with dynamic colpocystorectography [13]. However, there are an increasing number of reports in which VD is considered to be superior to MRD in the imaging of defaecation disorders [14, 15, 16]. There is a proven risk that enteroceles and internal and external rectal prolapses might have been missed by MRD in patients with ODS or FI [14, 15, 16]. Although the supine position in MRD is nonphysiological, only a few studies report the rate of unsuccessful straining or defaecation and the diagnostic capabilities of MRD [2, 14, 17].

Considering the contradiction in previously published reports on VD and MRD in the imaging of defaecation disorders, our aim was to compare the findings of these two methods and analyse the technical success rates.

## METHOD

### Study design

This retrospective study includes women who underwent both VD and MRD in our hospital between the years 2007 and 2017. We excluded cases in which the interval between the two studies was more than 1 year. We also excluded women who had undergone pelvic surgery after the first examination (VD or MRD).

### Video defaecography

Before the examination, the patient received written instructions for the study. Verbal instructions were given at the time of the study by both the technician and the radiologist in charge. Before the examination, the rectum was emptied by a water enema. Opacification of the small bowel was achieved by drinking 500 ml of barium solution (Mixobar Colon® 1 g/ml or Liquid Polibar Plus® 1 g/ml 250 ml + water 250 ml) 1 h before the examination. VD was performed using Artis Zee (Siemens) fluoroscopy equipment. With the patient lying on her side, the rectum was filled with 300 ml of gel that was made in our hospital pharmacy. The gel was a semisolid mixture of Liquid Polibar Plus® 1 g/ml, ultrasound gel, methyl cellulose and 85% glycerol. The vagina was marked with 10 ml of a mixture of Omnipaque® and ultrasound gel. After that, the patient was placed in a commode and seated sideways. Fluoroscopy started at a rest stage after which the patient was asked to squeeze. After squeezing the patient was asked to strain and evacuate. Straining and evacuation were repeated until it was evident that proper straining/emptying was reached.

### MR defaecography

The patient received written instructions with the invitation letter. The course of the examination was carefully repeated by the technician in charge when the patient arrived at the MRI department. MRD was performed using a 1.5 T magnet (Optima, General Electric). Before the study, the rectum was filled with 200 ml of gel (Resource® Thickenup® instant thickener, Nestlé) through a rectal catheter. A phased array coil was used while the patient was lying supine in the magnet with knees slightly bent. A FIESTA sequence was obtained (TR 5.2 ms, TE 2.1 ms, matrix 256 × 256, field of view 33 cm, slice thickness 6 mm). A midsagittal plain was defined and dynamic images were obtained during a squeeze, after which the patient was asked to strain and evacuate. Images were repeatedly (up to five times) obtained during straining and evacuation until it was evident that proper straining/evacuation was reached. Each dynamic sequence lasted 54 s.

### Image analysis

Both studies were analysed in consensus by two radiologists (VD by senior radiologists EP and HO with more than 20 years' experience of body radiology and MRD by EP and HL, who is a young certified radiologist). The time interval between analysing the images was at least 1 year. The radiologists were blinded to clinical patient data and radiology reports.

The technical success rate (T) was analysed visually by assessing straining and evacuation on a scale from 1 to 5, as follows:

T1: adequate straining and evacuation. Clear pelvic floor movement was observed with evacuation of all or most of the rectal content.

T2: adequate straining with deficient evacuation. Clear pelvic floor movement was observed with some evacuation but most of the rectal content was retained.

T3: adequate or partial straining with no evacuation. Pelvic floor movement was considered normal or partial but all rectal content was retained.

T4: no straining or evacuation. No pelvic floor movement was observed and none of the rectal content was evacuated.

T5: incontinence.

The diagnostic success rate (D) of the images was classified on a scale from 1 to 3, as follows:

D1: fully diagnostic, including all T1 cases.

D2: partially diagnostic, including T2 and T3 cases where dyssynergic defaecation or rectocele was observed but were nondiagnostic considering enterocele and most cases of internal and external prolapse.

D3: nondiagnostic, including all T4 cases together with some T3 cases where movement of the pelvic floor was deficient, not allowing any diagnostics.

The imaging findings were analysed using established interpretation criteria, paying special attention to enterocele, peritoneocele, internal (recto‐rectal or recto‐anal) and external rectal prolapse, rectocele (≥2 cm anterior bulge) and dyssynergic defaecation [18, 19]. The study was approved by the institutional review board.

### Statistical analyses

Summary statistics are presented as mean with standard deviation (SD) unless otherwise stated. The McNemar test was performed to compare VD and MRD in their ability to make a full diagnosis. The kappa coefficient and intracorrelation coefficient (ICC) with 95% confidence intervals (95% CI) were calculated to describe the consistency of diagnoses between VD and MRD. The kappa coefficient was calculated for categorical data and the ICC for continuous data. Kappa and ICC values are interpreted as follows: <0.20 represents slight reliability, 0.21–0.40 represents fair reliability, 0.41–0.60 represents moderate reliability, 0.61–0.80 represents substantial reliability and >0.80 represents almost perfect reliability. Analyses were performed using SPSS for Windows (IBM SPSS Statistics for Windows, version 25.0, IBM Corp., released 2017).

## RESULTS

### Patient characteristics

The study included 64 women (mean age 56 years, range 26–85 years) who underwent both VD and MRD within a year. A total of 1324 VD and 466 MRD examinations were performed in our hospital during the years 2007–2017. Our patient population represents 4.8% of VD and 13.7% of MRD examinations performed during that time. The interval between the two studies was 4–363 days (mean 158 days). There were 24 patients who had undergone hysterectomy before the first examination. MRD was the first examination in 35 patients, whereas VD was the first examination in 29. In 58 patients, symptoms of obstructive defaecation were the indication for the first study, while six patients had incontinence as the primary diagnosis. The indication for the second study was insufficient information from the first study (either technical failure or the findings did not correlate with the clinical status or the patient's symptoms) in 48 patients. In 16 cases, the second imaging was performed before operative treatment to get more anatomical information or to confirm the findings of the first study.

### Imaging

The technical success rate and diagnostic capabilities of VD and MRD are presented in Table 1. Of the 64 patients, 62 (96.9%) reached adequate strain and evacuation in VD, making the study fully diagnostic. In one case with incontinence in VD, enterocele was observed and the study was considered partially diagnostic. On the other hand, 29/64 (45.3%) patients reached adequate strain and emptying in MRD to allow reliable diagnostics (*p* < 0.001, McNemar). In 21/64 (32.8%) patients, MRD was considered partially diagnostic, which allowed evaluation of pelvic floor muscular dyssynergy and rectocele. In addition, in the group of partially diagnostic studies, evacuation was good enough to diagnose three cases of recto‐rectal intussusception (one T2 and two T3). In 14/64 (21.9%) patients, MRD was considered nondiagnostic due to lack of or insufficient straining and evacuation.

**TABLE 1:** Technical success (T) and diagnostic capabilities (D) of VD and MRD in 64 patients

	VD	MRD
Straining and evacuation		
T1: adequate	62 (96.9%)	29 (45.3%)
T2: adequate straining, partial evacuation		10 (15.6%)
T3: adequate or partial straining, no evacuation		16 (25%)
T4: no straining or evacuation	1 (1.6%)	9 (14.1%)
T5: incontinence	1 (1.6%)	
Diagnostic capabilities		
D1: fully diagnostic	62 (96.9%)	29 (45.3%)
D2: partially diagnostic	1 (1.6%)	21 (32.8%)
D3: nondiagnostic	1 (1.6%)	14 (21.9%)

Abbreviations: MRD, magnetic resonance defaecography; VD, video defaecography.

**FIGURE 1:**
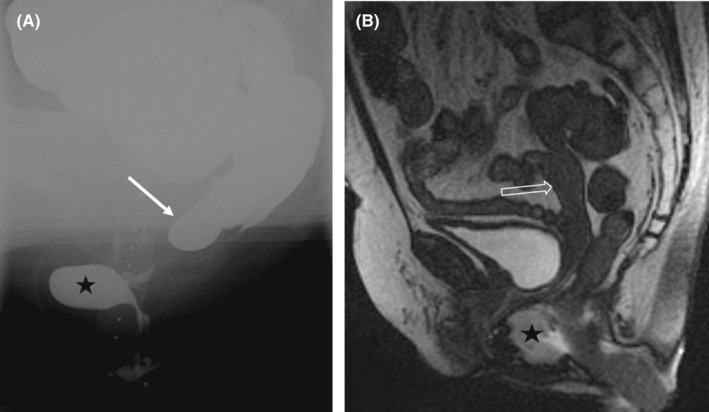
In a patient with obstructive defaecation syndrome, video defaecography (A) shows a typical enterocele (arrow) which is not visible in MR defaecography 4 months later (B). A rectocele (star) is observed in both studies. The uterus (open arrow) is seen in MR defaecography (B)

Enterocele was diagnosed in 30 patients by VD and in seven patients by MRD (Table 2, Figure 1). In one case, a peritoneocele was observed by MRD in a patient who had enterocele on VD. The degree of agreement was moderate (*κ* = 0.41). Recto‐rectal or recto‐anal intussusception was observed in 53 patients by VD (22 recto‐rectal and 31 recto‐anal) and 27 patients by MRD (24 recto‐rectal and 3 recto‐anal). Two external rectal prolapses were observed by VD whereas in both cases MRD was nondiagnostic. The agreement between the two methods was poor in diagnosing intussusception (*κ* = −0.10 and *κ* = 0.02 in recto‐rectal and recto‐anal intussusception, respectively). Dyssynergic defaecation was a more frequent finding in MRD compared with VD, at 11 and 3 cases, respectively. Moderate agreement was observed in diagnosing rectocele (*κ* = 0.47) between VD and MRD, with 47 and 29 cases, respectively. The mean rectocele size was 42 mm with VD and 33 mm with MRD, with moderate agreement (ICC = 0.55).

**TABLE 2:** Imaging findings in VD and MRD in 64 patients

Finding	VD (*n* ^a^/ n^b^)	MRD (*n* ^a^/ *n* ^b^)	Kappa^c^/ICC^d^ (95% CI)
Enterocele	63/30 (46.9%)	29/7 (12.5%)	0.41^c^ (0.09 to 0.73)
Peritoneocele		29/1 (1.6%)	n.d.
Intussusception			
Recto‐rectal	62/22 (34.4%)	32/24 (37.5%)	−0.10^c^ (−0.37 to 0.17)
Recto‐anal	62/31 (48.4%)	32/3 (4.7%)	0.02^c^ (−0.28 to 0.31)
Rectal prolapse, external	62/2 (3.1%)		n.d.
Dyssynergic defaecation	63/3 (4.9%)	50/11 (17.2%)	0.14^c^ (−0.35 to 0.62)
Rectocele	63/47 (71.9%)	40/29 (45.3%)	0.47^c^ (0.14 to 0.80)
Rectocele size (mm), mean (SD) [min–max]	42 (12) [25–70]	33 (9) [20–51]	0.55^d^ (0.21 to 0.77)

Abbreviations: CI, confidence interval; ICC, intracorrelation coefficient; MRD, magnetic resonance defaecography; VD, video defaecography.

^a^ Number of technically successful studies.

^b^ Number of diagnoses (percentage of diagnoses among technically successful images).

^c^ Kappa.

^d^ ICC.

## DISCUSSION

Video defaecography and MRI are widely used in imaging of patients with defaecation disorders. However, little attention has been paid to the success of straining and evacuation, which are essential in diagnosing pathology. A consensus statement was published recently underscoring the importance of rectal emptying for an assessment of the completeness of the examination [20]. This is of concern especially in MRD where the nonphysiological supine position may not allow sufficient straining and evacuation. In our retrospective study, as many as 35/64 (54.7%) MRD cases were not diagnostic for enterocele and 32/64 (50%) were not diagnostic for external or internal rectal prolapse. There are only a few previous studies reporting the rate of inadequate examinations in MRD. In those reports, the rate of inadequate MRD varied from 4.4% to 28.6% [2, 11, 14, 16, 17, 21]. The highest rate of technically inadequate MRD was reported by Pilkington et al. [14]. In their report, 11/35 cases of intussusception were not visible by MRD due to failure of rectal evacuation in 10 patients (28.6%). On the other hand, a 100% success rate was reported by Martín‐Martín et al. [22]: in all 40 patients, defaecation was satisfactory. However, they reported five cases of dyssynergic defaecation, which raises the question of the true diagnostic success.

The reasons for our poor technical success in MRD remain unclear. Most of our patients had ODS (58/64, 91%) which may partly explain poor defaecation. However, in the study by Piloni et al. [17], in which all 105 patients had ODS, only 9.5% of examinations were unsuccessful. The mean age of our patients was 56 years, compared with 46.1–64.3 years in other studies [11, 16, 17]. It is probable that there are factors other than ODS and patient age to explain our results. It is already widely accepted that examinations of defaecation are embarrassing for patients and the position in MRD is not physiological. Less attention has been paid to the closed environment and noise in MRI, which may further embarrass the patient and make defaecation even harder. Patients cancelling before their examinations is an extremely important factor [23]. In spite of written and verbal instructions before MRD, the miss rate was very high in our study. MR departments are usually very busy, and it remains unclear how much time the technicians have to prepare each patient for the examination.

Our results were also poor considering the diagnostic findings in MRD compared with VD. Agreement between the two methods was moderate in diagnosing enterocele and rectocele (*κ* = 0.41 and 0.47, respectively) but poor or slight in intussusception (*κ* = −0.10 in recto‐rectal and 0.02 in recto‐anal intussusception) and dyssynergic defaecation (*κ* = 0.14). VD revealed 30 enteroceles and 53 intussusceptions compared with 7 and 27 in MRD, respectively. High miss rates of enterocele (42.9%–83%) and intussusception (31%–36%) have also been reported in other studies with a limited number of patients [14, 16, 24]. These findings are strikingly different from reports where MRD was even better than VD, or at least as good as it, in diagnosing enterocele and intussusception [11, 12, 13, 22]. A conclusion was made that MRD could become the method of choice for evaluating ODS [22]. Excellent results were achieved comparing MRD with surgery and clinical examinations in a study of 26 patients where a sensitivity of 73%–100% was reached for enterocele and 86%–100% for rectal invagination [25]. No comparison with VD was performed in that study.

Despite the conflicting results, there is growing evidence that MRD in patients with defaecation disorders may underdiagnose many relevant findings, such as enterocele and intussusception. In a systematic review and meta‐analysis, MRD was no better than fluoroscopy in any outcome of interest [26]. It was pointed out that clinicians have to be mindful of the risk of underdiagnosis when using MRD in pelvic floor dysfunction.

In their appropriateness criteria, the American College of Radiology consider fluoroscopy as the method of choice for imaging patients with defaecatory dysfunction, and it has been emphasized that fluoroscopy is a practical and cost‐effective procedure for the evaluation of anorectal and pelvic dysfunction [27, 28]. There remains a concern about the availability of proper equipment for fluoroscopic imaging because traditional fluoroscopy studies have been replaced by CT and MRI, and the amount of fluoroscopy equipment is decreasing accordingly. Fluoroscopy carries the potential risk of ionizing radiation, especially in younger patients. However, with modern fluoroscopy equipment, the dose of radiation can be reduced to lower the potential risk. With optimization, a dose estimated to be as low as 0.3 mSv for VD has been achieved in our hospital. This is remarkably lower than the dose of 4.9 mSv reported in 1990 [29].

There are a number of limitations in our study. When interpreting and comparing the results of our study, we note that the need for additional imaging often arose from inadequacies in the first study. MRD was performed first in 35 patients and VD in 29 cases. This may partly explain the higher missing rates that we report in contrast to VD versus MRD studies carried out in prospective settings. However, as such, this study describes ‘real‐life policy’ in diagnostic imaging among this patient group. Due to the retrospective nature of the study, the interval between VD and MRD varied from 4 days to a year, with a mean of 5.3 months. However, the symptoms of defaecatory dysfunction are usually long‐standing and it can be presumed that the symptoms persisted or did not improve because a second study was ordered. The amount of rectal filling was 300 ml in VD compared with 200 ml in MRD, which may have influenced the worse emptying in MRD. Finally, the consistence of the gel in both instances was semisolid but was not compared directly, so it remains unclear if the consistency influenced the emptying of the rectum.

The analysis of strain and defaecation was subjective in our study. In a joint recommendation, MRD is considered diagnostic if a clear movement of the abdominal wall is seen during squeezing and straining [8]. However, we paid visual attention to the movement of pelvic floor and opening of the anorectal angle. Rectal emptying was also subjectively assessed and no quantification was performed in VD as recommended in a consensus [20]. A further limitation of our study is that there was no consistent control of bladder repletion as recommended [8]. We did not pay attention to the anterior and middle compartments of the pelvic floor. Because bladder contrast was not used in VD, other findings, such as cystocele, which may affect rectal emptying were not analysed.

We considered MRD to be an attractive method because no ionizing radiation is used and it offers a comprehensive view of pelvic soft tissues. Good published results have also been obtained when comparing it with VD [11, 13]. However, based on our growing experience, the number of MRD examinations has decreased in our hospital. Between the years 2013 and 2020, the decrease was 47% (from 49 to 26). In spite of its disadvantages, there is still a role for MRD in certain cases. It can be used as the first imaging method in younger patients with defaecation disorders. Because of superior soft tissue contrast it can be used in cases where anatomical details are critical, such as the anatomy of the levator ani and sphincter muscles. Patients with multicompartmental prolapse may also benefit from MRD [8]. MRI is the only method that can visualize postoperative meshes, such as those with MRI‐positive markings applied during ventral rectopexy [30]. MRD may also be used in those rare cases where VD is nondiagnostic.

## CONCLUSIONS

In this retrospective study of functional imaging in patients with defaecatory dysfunction, MRD was inferior to VD technically and by diagnostic performance. Radiologists and clinicians have to be mindful of the performance of the methods, both of which have a role in the imaging of patients with pelvic floor insufficiency.

## CONFLICT OF INTEREST

There are no conflicts of interest.

## Data Availability

Research data are not shared.
